# Experimental and Theoretical Study of Fluorescent Properties of Morin

**DOI:** 10.3390/molecules27154965

**Published:** 2022-08-05

**Authors:** Alexandra Deriabina, Tatiana Prutskij, Leticia Castillo Trejo, Maria Patricia Sanchez Gutierrez, Eduardo Gonzalez Jimenez

**Affiliations:** 1 Faculty of Physical and Mathematical Sciences, Autonomous University of Puebla (BUAP), Puebla 72570, Mexico; 2 Sciences Institute, Autonomous University of Puebla (BUAP), Puebla 72570, Mexico

**Keywords:** morin, fluorescence, TDDFT, ESIPT

## Abstract

Morin (**M**) is one of the most widely distributed flavonoids with several beneficial effects on human health, and has the potential of being used as a possible treatment for COVID-19. To achieve a better understanding of the process of **M** dissolution, the fluorescent (FL) emission from **M** solutions prepared with different polar and nonpolar solvents (methanol, DMSO, and chloroform) was measured and compared with the FL emission from **M** powder and **M** crystals. In the FL spectra of the solutions with high **M** concentration, as well as in the spectra of **M** in solid state, two features, at 615 nm and 670 nm, were observed. As the solution concentration decreases, the maxima of FL spectra of the **M** solutions in all considered solvents shift to the blue side of the spectrum until reaching the value of 520 nm. To explain the experimental results, the TDDFT-M06-2X/6-31++G(d,p) method was used to determine the possible electronic transitions in the **M** molecule. The computations show that the FL emission in the spectral range of detection of our setup (405–800 nm) is related to the excited state intramolecular proton transfer (ESIPT). Comparison of the experimental data with the computations strongly suggests that in low-concentrated solutions, the FL emission is mostly due to electronic transitions in the keto OH3 form, whereas in aggregated states, the dominate contribution to the FL emission spectra is due to the transitions in keto OH5 form. Moreover, the time evolution of the **M** solutions FL spectra was observed, measured and explained for the first time.

## 1. Introduction

**Figure 1:**
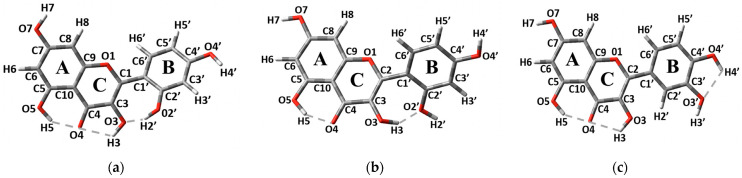
Two molecular configurations of **M** reported in crystals of MH [4] MCA (**a**), MCB (**b**) and the structure of the Q molecule (**c**). A, B and C are labels typically used for flavonoid molecule’s rings.

Morin (**M**), 3, 5, 7, 2′, 4′-pentahydroxyflavone is one of the most widely distributed flavonoids found in a variety of plants, such as branches of white mulberry, almonds, oranges, fig fruits, mill, old fustics, sweet chestnuts, onions, apples, leaves of guajava, etc. [1,2,3]. Usually, **M** is present in solid phase as Morin Hydrate (MH) where two different molecular configurations, molecular configuration A (MCA) and molecular configuration B (MCB), are reported (Figure 1a,b) [4]. **M** exhibits a variety of types of pharmacological activities, such as free radical scavenging, anti-inflammatory action, xanthine oxidase inhibitor property, protective action on DNA from damage caused by free radicals, prevention of low-density lipoprotein oxidation, anti-cancer activity, etc. [1,5,6,7,8,9]. New studies show that **M** also has a high potential to be used as a possible treatment for COVID-19 [10,11]. **M** is a very promising compound, having some advantages over another flavonoid, Quercetin (Q), 3,5,7,3′,4′-pentahydroxyflavone (see Figure 1c). Q is more widely represented in nature and is commonly used as a food supplement. It was shown in rats that the bioavailability of **M** is higher than that of Q. Additionally, the response to the higher doses of **M** is superior to that of Q [12]. This suggests that a small change in the molecule configuration, i.e., the change of the OH group’s position from 3′ in Q to 2′ in M, can lead to a noticeable increase in the compound’s bioavailability. Nevertheless, the **M** bioavailability and, therefore, its practical relevance are limited because of its low solubility.

Optical properties of **M** solutions were studied previously by several authors to achieve a better understanding of the **M** dissolution process. Absorption and fluorescent (FL) emission of **M** methanol–water mixtures with different water proportions were studied in reference [13]. A wide FL peak with a maximum at approximately 490 nm was observed in the FL spectra of **M** solutions in methanol. The increase in the water content of the solution has induced the red-shift of the FL emission peak and a noticeable decrease in its intensity. If acetonitrile was used instead of methanol, two FL peaks at 500 and 560 nm were observed. Yu et al. [14] studied the FL emission spectra of the **M** solutions with a concentration of 50 μM, in pure DMSO and in DMSO with different amounts of added water. The FL emission spectra of **M** solutions in pure DMSO contained the FL peak with a maximum at 560 nm and a shoulder at 500 nm. The increase in the water amount of the **M** solution has led to a lower intensity in the FL peaks, without changing their spectral position. The FL emission of **M** aqueous solutions with a low **M** concentration having different values of pH was studied in reference [15]. To obtain the aqueous solutions of **M,** it was first dissolved in methanol. It was found that the maximum of the FL emission peak of the **M** solution shifted from 525 to 560 nm when increasing the pH of the solution.

In all these papers, the solutions with low to moderate **M** concentrations were studied; however, as far as we know, neither solutions with moderate to high **M** concentrations, nor the spectral position of the FL peaks as a function of the **M** solution concentration have been considered yet. Additionally, to the best of our knowledge, the computation results were only presented for calcium–**M** systems [15].

The present work is closely linked to our previous study of the dependence of the FL emission peak spectral position on the Q concentration in the solution [16]. In that paper, we studied how the FL emission of Q molecule changed with the variation of the concentration of solutions in different types of solvents. Our study showed that the FL spectra presented a noticeable spectral shift of the FL emission peaks when the Q concentration was decreased, and that this shift is similar for all three types of used solvents (polar protic, polar aprotic and nonpolar). In this paper, we perform a similar study of the **M** FL spectra, also addressing the issue of the time evolution of the FL emission of **M** solutions, and show that the FL spectra can be used to study the process of **M** dissolution.

This paper is organized as follows: after an introduction given in Section 1, Section 2 exposes images of the **M** crystals and displays the FL spectra of **M** in its solid state, and of **M** solutions prepared with different solvents. Then, the results of computation of the characteristic wavelengths are shown. In Section 3, computational results are discussed and compared with that obtained experimentally. In Section 4, the materials and experimental procedure are described together with the computational details. Conclusions are given in Section 5.

## 2. Results

### 2.1. Fluorescent Properties of Morin

The luminescent characteristics of **M** in its solid state (powder and crystals), and of **M** solutions prepared with different solvents were studied and compared to each other.

#### Morphology of Morin Crystals. FL Spectra of M Crystals and Powder

**Figure 2:**
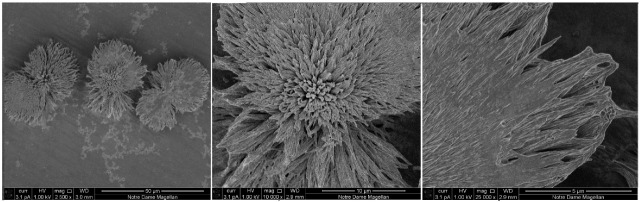
SEM images of the **M** crystals shown with different magnification.

**Figure 3:**
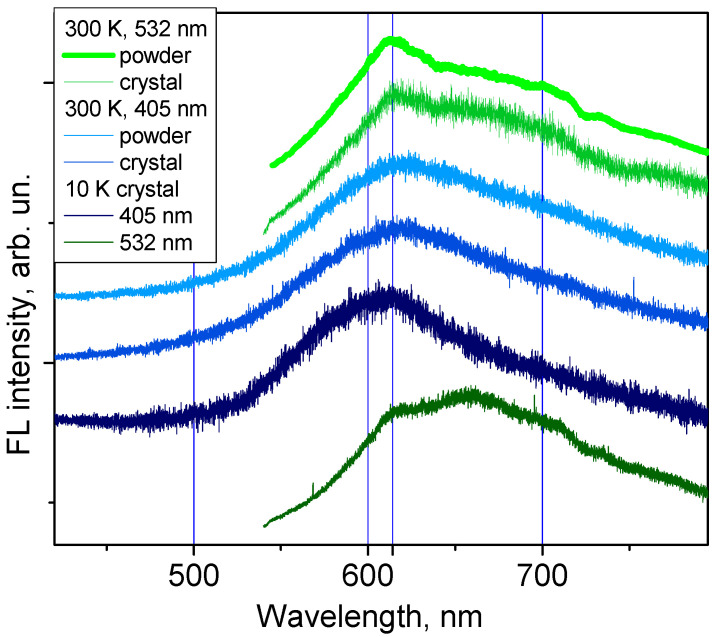
FL emission spectra of MH powder and **M** crystal at 300 K, and of **M** crystal at 10 K measured with two different excitation wavelengths (blue curves for 405 nm and green curves for 532 nm). To facilitate comparison the spectra are shown in arbitrary units.

Crystals of **M** were obtained at room temperature from the MH powder saturated solution in methanol. In Figure 2, **M** crystal images obtained by scanning electron microscope (SEM) are shown. **M** crystals are needle-like, sharp crystals, very similar to that of Q obtained by the same method and studied in our previous work [16]. The FL spectra of the crystals are shown in Figure 3, and are compared with the spectra obtained on MH powder. MH powder was measured as received from the supplier, i.e., without any additional treatment.

To study FL properties of the **M** solid state, we used two different excitation wavelengths: 405 nm and 532 nm. At room temperature, the FL spectra obtained on **M** crystals and MH powder are very similar, reaching their maximum at approximately 615 nm; they are also similar to the spectra of Q shown in reference [16]. To our surprise, the FL spectrum at 10 K does not show significant changes. When excited by the light with the wavelength of 532 nm, the spectrum of the **M** crystal at 10 K showed the same FL peak at 615 nm and revealed the feature at 670 nm. Furthermore, at low temperature, the full width at half maximum (FWHM) of the spectrum does not reduce significantly.

### 2.2. FL Spectra of Morin Solutions

Solubility of **M** defines its potential to interact with other molecules and, therefore, its use as a potential drug, as well as the drug’s dose. To understand the role of the solvent in **M** solutions, three different types of solvents were used: methanol as the polar protic, dimethyl sulfoxide (DMSO) as the polar aprotic and chloroform as the nonpolar solvent. The solubility of **M** in the nonpolar solvent is less than in the polar one and, therefore, the time needed to dissolve the MH powder in chloroform was significantly greater than that used for the preparation of solutions based on polar solvents. As far as we know, there is no data about **M** solubility in chloroform; therefore, we first prepared a saturated solution of **M**, and then the upper part of that solution was separated and dissolved several times in order to make solutions with different concentrations. The fact that the solubility of **M** in polar solvents is better than in the nonpolar one points out to the considerable value of the dielectric moment of **M** molecule. On the other hand, its dissolution in water is very low. The same was observed on Q solutions [16].

Further, we observed that the FL spectra of all solutions were changing with time, this effect being greater for solutions with low **M** concentrations: the spectral maximum of the **M** solution spectrum slowly shifts with time towards the blue side of the spectra until reaching the value of 520 nm, and then it remains stable. We observed the same time dependence of the FL emission peak spectral position for different solutions of Q [16].

**Figure 4:**
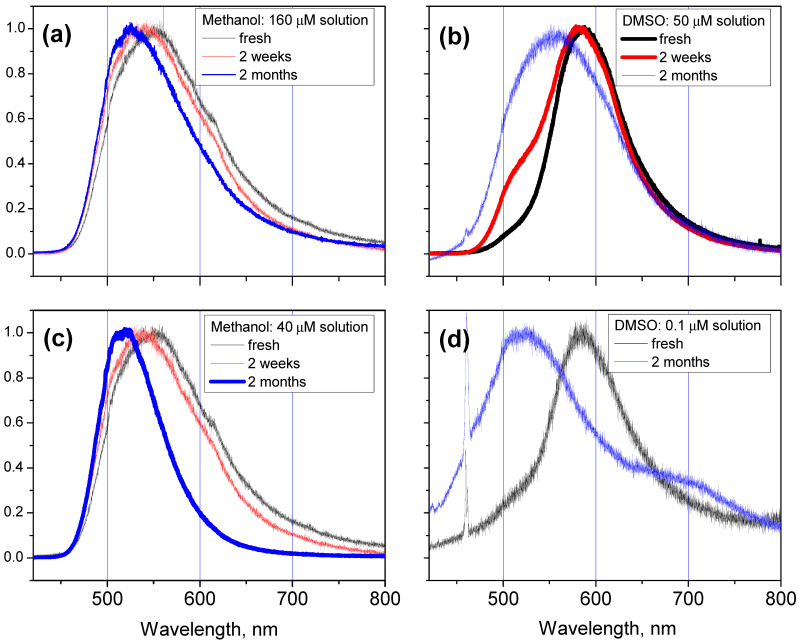
Time dependence of the FL spectra of **M** solutions in methanol (**a**,**c**) and DMSO (**b**,**d**). The spectra of solutions with moderate (**a**,**b**) and low (**c**,**d**) **M** concentrations are shown. All the spectra were normalized to one by dividing each spectrum by its highest value. The sharp peaks in (**d**) at approximately 460 nm are due to the emission of DMSO solvent.

The evolution with the time of the FL emission spectra of **M** solutions in methanol (a, c) and in DMSO (b, d) with different concentration is shown in Figure 4. We show the FL spectra of **M** solution with a concentration of 40 μM in Figure 4c, since all the spectra of aged **M** solutions in methanol with the concentration lower than that value have the same spectral shape and position, and differ only in their intensity. Moreover, the FL spectrum of low-concentrated aged **M** solution in DMSO (see blue curve in Figure 4d) shows a spectral feature at 670 nm that was also detected in the FL spectrum of the **M** crystal.

**Figure 5:**
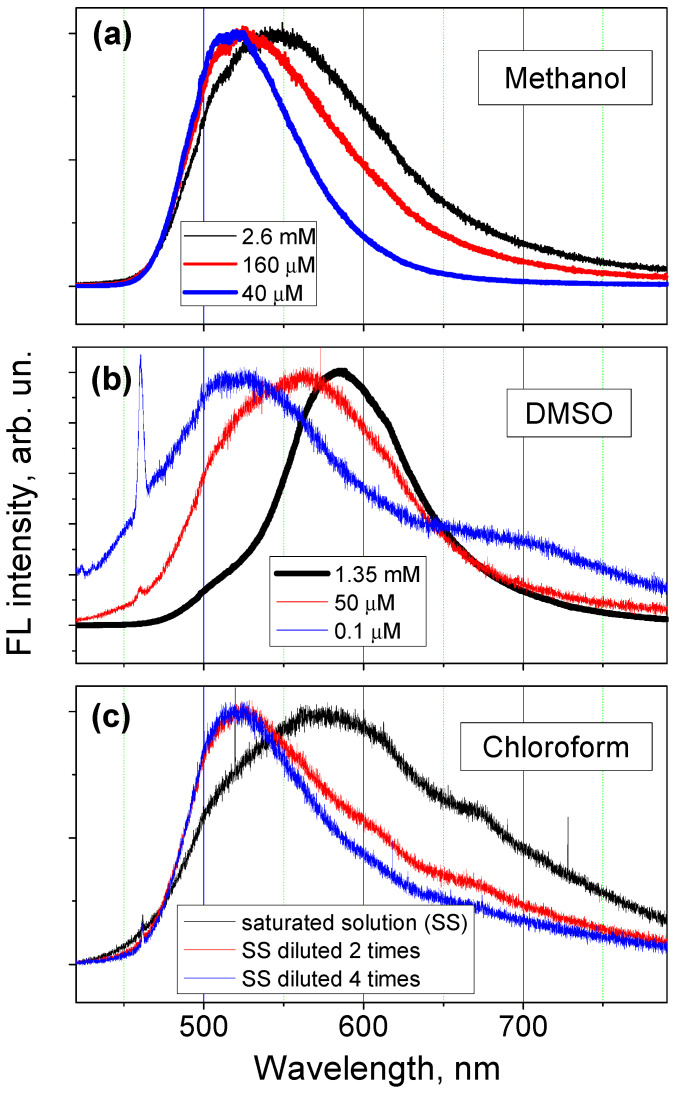
FL emission spectra measured at room temperature on **M** solutions in: (**a**) methanol, (**b**) DMSO, and (**c**) chloroform, with different concentrations. The sharp emission peak in (**b**) at approximately 460 nm is due to the emission of DMSO solvent. To facilitate comparison, all the spectra were normalized to one by dividing each spectrum by its highest value.

The FL spectra of **M** solutions with different concentrations are shown in Figure 5. The same FL spectrum of low-concentrated **M** solution in methanol is shown in Figure 4c and Figure 5a. The same FL spectrum of low-concentrated **M** solution in DMSO is shown in Figure 4d and Figure 5b. All the **M** solutions were measured after a time long enough to reach a stable spectral position of the FL spectrum. The solution stabilization process normally takes approximately 2 months. The FL spectra of **M** solutions have only one wide peak with its maximum located at 550–580 nm for high-concentrated solutions, which is close to the characteristic wavelengths observed on the FL spectra of the **M** solid state. Spectra of the low-concentrated solutions in all selected solvents are narrower and their maxima are shifted to the blue side of the spectrum. It can be seen that the FL spectra of low-concentrated **M** solutions in the considered solvents are similar: all of them have their maxima at 520 nm and similar values of FWHM.

### 2.3. Computation of FL Characteristic Wavelengths

The molecular structure of **M** is similar to that of the Q molecule: it has two co-planar rings, A and C, and a B-ring, which can be rotated around the C2-C1′ single bond (see Figure 1). However, the change in the hydroxyl group’s position from 3′ to 2′ in **M** molecule removes the hydrogen bond (H-bond) in the B ring, and leads to the formation of the H-bond between the rings B and C in both molecular configurations, MCA and MCB, of the MH crystal [4]. O2′-H2′…O3 H-bond is formed in the MCA, while −OH3 hydroxyl group is rotated 180° in the MCB to form O3-H3…O2′ H-bond. The importance of the H-bonds in crystal structures is described in [17]. Both the MCA and MCB are noticeably non-planar having −43.4° (for MCA) and 51.0° (for MCB) torsion angles around the C2-C1′ bond [4]. All these characteristics require the use of a DFT functional capable of reproducing both the non-planarity of the molecule and the H-bond formation; therefore, the functional M06-2X was chosen. Additionally, the use of this functional provided a good agreement with the experimental data reported in our previous paper [16].

**Figure 6:**
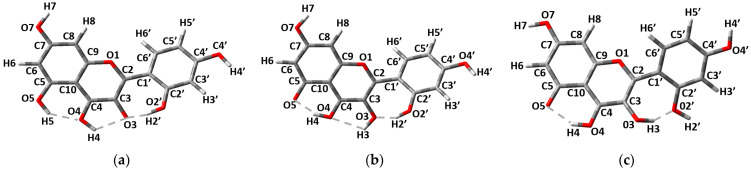
Keto forms of **M**: MCA in keto OH3 form (**a**), MCA in keto OH5 form (**b**), MCB in keto OH5 form (**c**).

Since the molecular structure of **M** is very similar to that of Q, its FL emission can also be attributed to the excited state intra-molecular proton transfer (ESIPT) [16,18,19,20,21]. As in reference [16], we will use the terms: “enol” for the molecular structures in Figure 1a,b; “keto OH3” for the tautomer form with H3 proton transferred towards O4 atom forming hydroxyl group –OH4 (Figure 6a); and “keto OH5” for the tautomer form with transferred H5 proton (Figure 6b,c). As can be seen in Figure 6, both keto OH3 and keto OH5 forms are possible for the MCA, while only the keto OH5 form exists for MCB. Consequently, the MCA was considered for all solvents, while for MCB only the computations in methanol and in vacuum were performed. Our computations show that, even with relatively strong changes in the distribution of H-bonds in **M** molecular configurations, the FL emission wavelengths do not change significantly (the difference is less than 25 nm). In Table 1, our computational results for absorption and emission wavelengths for the enol and keto forms of the MCA and MCB (see Figure 1 and Figure 6) in vacuum and in solvents are shown.

The calculated values of the absorption wavelengths for **M** enol forms are 299–310 nm in vacuum and 311–316 nm in all solvents. Thus, using the wavelength of 405 nm for excitation, we did not observe the FL emission due to the **M** enol electronic transition, but only the emission due to **M** keto forms. For the Q enol transition, the absorption wavelengths are 301 nm in vacuum and 328–329 nm in solvents [16]; so, the absorption in solutions for enol forms of **M** and Q molecules is red-shifted with respect to the absorption in vacuum. Additionally, the FL emission of the **M** enol form of MCA is very close to that of Q (λ_em_ in solvents is 381–395 nm for **M** vs. 381–388 nm for Q).

To estimate the characteristic wavelengths of absorption due to the electronic transition in keto OH5 form of MCA, we restricted the H5 proton position for the ground state, S_0_, since in the ground state the keto OH5 form does not has a local minimum. The corresponding values are shown in cursive in Table 1. Our computations show that both **M** keto forms of the MCA are stable in their excited states, S_1_, for all three solvents. The obtained values of the absorption wavelength due to the electronic transition in **M** keto OH3 form are of order of 399–433 nm, whereas the wavelengths due to the keto OH5 form are 386–404 nm. Therefore, both **M** keto OH3 and **M** keto OH5 transitions can be excited by the 405 nm laser line.

## 3. Discussion

### 3.1. Low-Concentrated Solutions

The PCM model, which considers one **M** molecule surrounded with implicit solvent, was used for the computations of the FL emission wavelengths in solutions. Thus, the computation results should describe better the experimental data obtained on low-concentrated solutions, i.e., on the solutions having the FL spectra with the greater shift towards the smaller wavelengths (see blue curves in Figure 5). For all the considered solvents, the values of calculated emission wavelengths are very close to each other for both **M** keto forms: the obtained values for the keto OH5 (OH3) are of 549–560 nm (475–478 nm) for the MCA and 537 nm for the MCB (for MCB keto OH3 does not exist). This finding is consistent with the fact that the FL emission peaks for different **M** solutions with low concentration have the same spectral positions (see Figure 5). The oscillator strength (f_em_) obtained for the electronic transition in the keto OH5 form of MCA is less than a half of that for the transition in the keto OH3 form. This indicates that the keto OH3 transition is more probable than that of keto OH5. On the other hand, the FL spectra shown in Figure 5 are broad, indicating that FL emission has several contributions; as a result, the FL emission corresponding to the keto OH5 transition with the maximum at approximately 550 nm can be covered by peaks with higher intensity.

The calculated values obtained for keto OH3 emission in methanol are also close to experimental data obtained in reference [13]. In that study, the FL emission peak at 495 nm for **M** solutions in methanol with concentration of 41 μM was reported. For **M** solutions in methanol with the same concentration, we obtained the FL emission peak at 520 nm. This difference in the spectral peak position can be attributed to the difference in excitation wavelengths (390 nm in reference [13] vs. 405 nm in this study).

### 3.2. Solid State

For calculations of absorption and emission wavelengths for **M** in solid state, the optimized molecule geometries starting from the enol forms of MCA and MCB were used (Figure 1a,b). During the geometry optimization in vacuum of the enol form of the MCB in the first excited state S_1_, the spontaneous H5 proton transition toward O4 oxygen takes place, and the electronic transition in the resulting keto OH5 form generates the FL emission at 581 nm. However, the enol form of the MCA presents a minimum in the first excited state S_1_ with the corresponding FL emission at 366 nm, that cannot be observe in our experimental setup. Nevertheless, the keto OH5 form of the MCA also exists having the minimum with the corresponding FL emission at 600 nm. Thus, for solids, the calculated FL emission wavelengths for the MCA and MCB in vacuum (considering the tautomer keto OH5 emission) are close to experimentally obtained values (615 nm vs. calculated 581–600 nm).

### 3.3. Moderate-High Concentrated Solutions

The FL spectra maxima of **M** solutions with moderate-high concentrations correspond to approximately 560 nm for all solvents. The spectrum of the **M** solution in DMSO with a concentration of 50 μM (see Figure 4b) is very similar to that reported by Yu et al. [14] (the spectrum of the **M** solution in pure DMSO in Figure 1b in reference [14]). These experimental values of 560 nm match the results of our calculations for the keto OH5 form’s radiative transition. On the other hand, this spectrum can be seen as the sum of two contributions: one of them due to the FL emission of the **M** molecule in solutions with low concentration described in Section 3.1, and another one due to the FL emission of the undissolved clusters of MH powder. The spectral position of the FL emission peaks of these clusters has to be close to that found for the solid state (see Section 3.2). The observed time-dependent blue shift of the FL emission peak shown in Figure 4 also could be explained by the fact that with time more **M** clusters are dissolved. Furthermore, in reference [14] it was also suggested that the FL emission with wavelength of 560 nm is related to the aggregation of **M** molecules.

## 4. Materials and Methods

### 4.1. Experimental Setup

A conventional experimental set-up, including a TRIAX550 monochromator and a liquid-nitrogen-cooled charge-coupled device (CCD) detector was used for recording the FL emission. Optical excitation was provided by a laser diode with a wavelength of 405 nm or by a solid-state laser with a wavelength of 532 nm. To avoid degradation, a low excitation light intensity of approximately 5 mW was used in all measurements.

Morin Hydrate (M4008 powder with >85% purity) was acquired from the Sigma-Aldrich. Methanol (>99.5%), DMSO (>99.60%), and chloroform (>99.8%) were purchased from J.T. Baker.

### 4.2. Computational Details

The density functional theory (DFT) [22] method was used for full geometry optimization and for vibrational frequency calculations of the **M** molecule. For computation of the excited states, the time dependent DFT (TDDFT) [23] approach was used via the Minnesota functional M06-2X [24] and 6-31++G(d,p) basis set [25], including polarization and diffuse functions implemented in Gaussian 16 [26]. The influence of the solvents (methanol, DMSO, chloroform) in the FL emission wavelength was simulated by using the polarizable continuum model (PCM) within the self-consistent reaction field (SCRF) method [27].

## 5. Conclusions

1. Crystals of **M** were fabricated from saturated **M** solutions and their morphology and FL emission spectra were measured and compared with the FL spectra of MH powder. The FL spectra obtained on **M** crystals and MH powder are very similar, having their maximum at approximately 615 nm.
2. The FL emission spectra of solutions of **M** in different types of solvents were obtained and compared with FL emission spectra of the **M** solid state. The spectral position of the FL maximum depends on the concentration of the solution: when the **M** concentration decreases, the FL spectra of the low-concentrated aged **M** solutions have a spectral blue shift up to 520 nm.
3. The FL emission spectra of **M** solutions in all solvents change with time: in fresh solutions the maxima of the FL emission peaks correspond to the longer wavelengths. With time, the FL spectra of low-concentrated solutions shift towards the blue side of the spectrum and settle at 520 nm. As far as we know, the effect of the change with time of the spectral position of the **M** solutions FL spectra was not reported before.
4. Computations performed using TDDFT-M06-2X/6-31++G(d,p) level of theory show that the keto OH3 form’s FL emission wavelengths (490 nm in vacuum, 475–478 nm in solvents) are significantly smaller than that found for keto OH5 form’s (581–600 nm in vacuum and 537–553 nm in solvents). In the computations for solvents, the PCM model that considers a molecule surrounded completely by the solvent molecules and, thus, suitable for comparing with the results obtained in to low-concentration solutions was used. The comparison of the experimental data with the computations strongly suggests that in aggregated states (as in solids or high-concentrated solutions), the keto OH5 form’s contribution to the emission spectra is the predominant one, while for low-concentrated solutions, the keto OH3 form’s FL emission dominates.
5. Both experimental results and theoretical estimations show that for all considered solvents, the difference between the characteristics wavelengths is small; moreover, earlier we obtained similar results for Q [16]. This shows that the results of this work can be extended to other solvents, as well as to other flavonoids.
6. Our study allows the establishing of a criterion of the flavonoid’s molecules dissipation in the low concentrated solutions: the molecules are dissipated when the FL emission peak of the solution is stable at its lowest wavelength.

## Figures and Tables

**Table 1:** Absorption and emission of MCA and MCB obtained at TDDFT-M06-2X/6-31++G(d,p) level of theory.

	E_S0_ (a.u) S_0_ opt	E_S1_(a.u) S_0_ opt	E_S1_-E_S0_ (eV)	λ_ab_ (nm)	f_ab_	E_S1_* (a.u) S_1_ opt	E_S0_* (a.u) S_1_ opt	E_S1_*-E_S0_* (eV)	λ_em_ (nm)	f_em_
MCA										
enol (Figure 1a)										
Vacuum	−1103.8349	−1103.6882	3.990	**310.60**	0.53	−1103.6991	−1103.8237	3.390	**365.71**	0.62
Methanol DMSO Chloroform	−1103.8526	−1103.7081	3.933	**315.29**	0.63	−1103.7200	−1103.8379	3.207	**386.68**	0.98
	−1103.8529	−1103.7087	3.923	**316.07**	0.65	−1103.7203	−1103.8382	3.206	**386.80**	0.98
	−1103.8477	−1103.7035	3.922	**316.19**	0.66	−1103.7142	−1103.8339	3.258	**380.62**	0.88
keto OH3 (Figure 6a)										
Vacuum	−1103.8153	−1103.7102	2.860	**433.45**	0.56	−1103.7170	−1103.8102	2.535	**489.08**	0.55
Methanol DMSO Chloroform	−1103.8364	−1103.7221	2.983	**398.72**	0.65	−1103.7327	−1103.8286	2.609	**475.22**	0.82
	−1103.8368	−1103.7225	2.976	**398.69**	0.67	−1103.7329	−1103.8289	2.611	**474.86**	0.83
	−1103.8304	−1103.7193	2.925	**410.13**	0.68	−1103.7283	−1103.8236	2.592	**478.35**	0.75
keto OH5 (Figure 6b)										
Vacuum	*−1103.8104*	*−1103.6973*	*3.080*	***402.91***	*0.24*	−1103.7137	−1103.7896	2.065	**600.47**	0.06
Methanol DMSO Chloroform	*−1103.8323*	*−1103.7142*	*3.213*	***385.90***	*0.35*	−1103.7353	−1103.8178	2.244	**552.60**	0.35
	*−1103.8327*	*−1103.7151*	*3.201*	***387.36***	*0.37*	−1103.7356	−1103.8186	2.257	**549.35**	0.36
	*−1103.8261*	*−1103.7134*	*3.066*	***404.41***	*0.35*	−1103.7299	−1103.8112	2.213	**560.37**	0.23
MCB										
enol (Figure 1b)										
Vacuum	−1103.8212	−1103.6689	4.147	**298.98**	0.31	-	-	-	-	-
Methanol	−1103.8476	−1103.7013	3.982	**311.40**	0.53	−1103.7148	−1103.8301	3.136	**395.46**	0.83
keto OH5 (Figure 6c)										
Vacuum	*-*	*-*	*-*	***-***	*-*	−1103.7064	−1103.7849	2.134	**581.07**	0.08
Methanol	-	-	-	-	-	−1103.7336	−1103.8184	2.307	**537.47**	0.37

Note: E_S0_ is the energy of the molecule optimized in the ground state S_0_; E_S1_ is the energy of the first excited state, S_1_, at the ground state optimized geometry, S_0_, from the non-equilibrium solvation state-specific calculation. For vacuum, E_S1_ is the energy of the first excited state, S_1_, at the ground state optimized geometry, S_0_; E_S1_* is the energy of the first excited state, S_1_, at its optimized geometry from the equilibrium solvation state-specific calculation. For vacuum E_S1_* is the energy of the first excited state, S_1_, at its optimized geometry; E_S0_* is the energy of the ground state, S_0_, with non-equilibrium solvation, at the optimized geometry of the excited state, S_1_. For vacuum E_S0_* is the energy of the ground state, S_0_, at the optimized geometry of the excited state, S_1_; f_em_ and f_ab_ are the oscillator strengths for emission and absorption, respectively.

## Data Availability

Data is contained within the article and can also be obtained from the corresponding authors.
